# Prediction of complex stimuli across saccades

**DOI:** 10.1167/jov.21.2.10

**Published:** 2021-02-23

**Authors:** Corinna Osterbrink, Arvid Herwig

**Affiliations:** 1 Department of Psychology and Cluster of Excellence Cognitive Interaction Technology, Bielefeld University, Bielefeld, Germany; 2 Department of Psychology, University of Bremen, Bremen, Germany

**Keywords:** peripheral perception, eye movements, prediction, complex stimuli

## Abstract

The visual system can predict visual features across saccades based on learned transsaccadic associations between peripheral and foveal input. This has been shown for simple visual features such as shape, size, and spatial frequency. The present study investigated whether transsaccadic predictions are also made for more complex visual stimuli. In an acquisition phase, new transsaccadic associations were established. In the first experiment, pictures of real-world objects changed category during the saccade (fruits were changed into balls or vice versa). In the second experiment, the gender of faces was manipulated during the saccade (faces changed from male to female or vice versa). In the following test phase, the stimuli were briefly presented in the periphery, and participants had to indicate which object or face, respectively, they had perceived. In both experiments, peripheral perception was biased toward the acquired associated foveal input. These results demonstrate that transsaccadic predictions are not limited to a small set of simple visual features but can also be made for more complex and realistic stimuli. Multiple new associations can be learned within a short time frame, and the resulting predictions appear to be object specific.

## Introduction

How is it possible that humans can recognize objects and people in their peripheral view even though retinal resolution is relatively low there? Answers to this question focus on the special role of previous experience gathered while moving the eyes (e.g., Stewart, Valsecchi, & Schütz, 2020; von Helmholtz, 1925). It has been suggested that these experiences are used by a transsaccadic prediction mechanism to facilitate peripheral object recognition (Herwig & Schneider, 2014). Accordingly, the visual system presaccadically predicts what an object will look like after we saccade toward it. To date, transsaccadic predictions have been shown to occur for simple visual features. However, to account for peripheral object recognition in a multitude of everyday situations, transsaccadic predictions need to occur also for more complex and natural stimuli.

### Peripheral object recognition and eye movements

Object recognition can occur when we fixate an object and thus have a foveal and therefore high-resolution image of it. But often, an object can be recognized even before we look at it, simply based on the peripheral image of it (e.g., Demeyer, De Graef, Wagemans, & Verfaillie, 2009; Kotowicz, Rutishauser, & Koch, 2010; Nuthmann, 2014). In a lot of scenarios, though, we make a saccade toward an object of interest and therefore end up seeing it presaccadically and coarse as well as foveally and detailed.

By performing a multitude of saccades, the brain learns to associate pre- and postsaccadic views of objects (e.g., Cox, Meier, Oertelt, & DiCarlo, 2005; Herwig & Schneider, 2014). These transsaccadic associations can be used to make predictions about what an object in the periphery will look like after a saccade toward it. It has been suggested that these predictions are integrated with the actual peripheral input prior to a saccade, thereby biasing our peripheral perception (Herwig, Weiß, & Schneider, 2015; Köller, Poth, & Herwig, 2020).

This has been experimentally investigated with a paradigm in which single and simple object features are changed during a saccade, thereby letting participants learn new transsaccadic associations. As a result, participants’ peripheral perception of these objects is biased. More specifically, for example, in the study by Herwig and Schneider (2014), participants had to look at two objects, differentiable by shape, containing a sinusoidal grating. One of these objects changed spatial frequency during a saccade (swapped object) and the other one stayed the same (normal object). Subjects learned these specific associations of peripheral and foveal object information for about 30 min. Following that, their perception of the spatial frequencies differed between the two objects. It was biased toward the previously associated foveal input. That is, participants for whom the spatial frequency of the swapped object changed from low to high perceived the frequency of the swapped object higher than that of the normal object. Equivalently, participants perceived the frequency of the swapped object lower if it had changed from high to low in the previous learning phase. Participants had learned to predict the spatial frequency of the object and integrated this prediction into their percept of the peripheral object, thereby biasing it.

Other studies have further investigated this transsaccadic learning and prediction. They could show that transsaccadic learning is not dependent on temporal or spatial object continuity (Weiß, Schneider, & Herwig, 2014) and does not require the actual performance of a saccade (Paeye, Collins, Cavanagh, & Herwig, 2018). How strongly the peripheral percept is biased by the prediction depends on the manipulated transsaccadic change size during acquisition (Köller et al., 2020).

Furthermore, transsaccadic learning and prediction have been shown with a variety of visual features like spatial frequency (Herwig & Schneider, 2014; Herwig, Weiß, & Schneider, 2018), size (Bosco, Lappe, & Fattori, 2015; Valsecchi, Cassanello, Herwig, Rolfs, & Gegenfurtner, 2020; Valsecchi & Gegenfurtner, 2016), and shape (Herwig et al., 2015; Köller et al., 2020; Paeye et al., 2018). The stimuli used in these studies were rather simplistic, and the manipulated features were ones that are usually classified as either low-level or mid-level in the visual system's processing hierarchy (e.g., Peirce, 2015; Ullman, Vidal-Naquet, & Sali, 2002). Integration of predictions into the visual processing of more complex and realistic stimuli, for which high-level semantic representations are built (e.g., Peirce, 2015), has not been investigated in detail yet. However, the latter are the kind of objects that we encounter in our natural environment. Thus, it is currently unknown whether transsaccadic learning and prediction also scale to more natural environments and govern peripheral object recognition outside the laboratory.

### Transsaccadic predictions for more complex stimuli?

The main aim of the present study is to investigate whether feature prediction is restricted to simple visual features or whether it occurs also for more complex and natural stimuli. Looking at existing literature, there are arguments in favor of each option.

There are some indications suggesting that transsaccadic predictions might be limited to simple visual features. First, the study by Herwig et al. (2018) could show that feature predictions are limited to their retinotopic location. They took this location specificity as evidence supporting the idea that feature prediction is incorporated into the visual perception in a retinotopically organized visual area, probably on a low or mid-level in the processing hierarchy (but see Valsecchi & Gegenfurtner, 2016).

Second, the location specificity of presaccadic feature prediction resembles perceptual learning, which is the training of a specific perceptual task resulting in a relatively permanent change—in most cases improved—performance (Fahle, 2004, 2005). Perceptual learning is argued to occur at least partly through changes in the primary visual cortex (Fahle, 2004) and is often specific to low-level features (Fahle, 2005). Possibly, the same restrictions could thus also apply for transsaccadic predictions.

On the other hand, there are also some indications suggesting that transsaccadic predictions could also be made for more complex stimuli. First, staying with their similarity to perceptual learning, there are studies that claim that perceptual learning can occur for both basic and complex stimuli (e.g., pictures of real objects) and that higher nonretinotopic cortical areas are involved in the process (Furmanski & Engel, 2000; Xiao et al., 2008).

Second, there is first evidence that transsaccadic learning is possible for more complex form changes. For example, in the study by Cox, Meier, Oertelt, and DiCarlo (2005), object identities of artificial greeble stimuli (Gauthier & Tarr, 1997) changed during the saccade toward a particular position, which induced object confusions across retinal positions.

Third, visual perception is typically biased toward recently seen stimuli—a phenomenon termed *serial dependence* (J. Fischer & Whitney, 2014). This has been shown for a variety of different simple stimuli such as letters, colors, and orientation, as well as more complex stimuli like faces (Kiyonaga, Scimeca, Bliss, & Whitney, 2017).

Last, considering the multitude of everyday situations, it seems reasonable that transsaccadic predictions, learned and updated throughout our lifetime, would also be created for real objects. These objects are usually not identified by a single feature dimension but are constituted of a complex variety of visual features. Thus, a mechanism that allows humans to better predict complex objects in the periphery is ecologically very useful.

### The present study

In the current study, we extended the experimental paradigm of Herwig and Schneider (2014) to investigate whether also complex visual features are predicted across saccades. As more naturalistic and complex stimuli, we used photos of fruits and balls (Experiment 1) and photos of faces (Experiment 2). Admittedly, this is still quite artificial input, but it is one step toward validating previous findings and gaining knowledge on underlying visual processing mechanisms that are functionally relevant in our everyday life (Felsen & Dan, 2005; Kayser, Körding, & König, 2004).

## Experiment 1: Balls and fruits

### Materials and methods

#### Aim

In the first experiment, we investigated if transsaccadic predictions can be learned for a set of pictures of real-world objects (fruits and balls). Half of the objects changed in category during the saccade (fruits changed to balls or vice versa), and half of them stayed the same in the acquisition phase. In the following test phase, participants were asked to identify the object they perceived in the periphery. We hypothesized that participants show increased error rates, specifically more category errors, with objects that were swapped out during the acquisition phase compared to objects that stayed the same during acquisition. Furthermore, we expected that they would incorrectly choose the associated object with a higher-than-chance rate. As an additional measure, participants also judged their subjective level of confidence, which can sometimes diverge from the decision accuracy (Kotowicz et al., 2010; Wilimzig, Tsuchiya, Fahle, Einhäuser, & Koch, 2008). It could be possible that newly acquired transsaccadic associations only lead to decreased confidence but are not reflected in the object choice. In this experiment, subjects learned six new associations. In previous experiments, only one new transsaccadic association had to be acquired by participants. Therefore, the experiment also tested whether multiple new transsaccadic associations can be learned in the span of the experiment.

#### Participants

There were 24 participants (17 female) who participated in Experiment 1. They were aged between 20 and 31 years (*M* = 24.29, *SD* = 2.61) and were students of Bielefeld University. All participants had normal or corrected-to-normal vision and were naive to the aim of the study. They received monetary reimbursement or course credits for their participation. The experiments were in accordance with the principles of the Declaration of Helsinki and approved by the local ethics committee. Written informed consent was obtained from the participants prior to the study.

#### Apparatus and stimuli

Experiments were conducted in a dimly lit room. Participants sat 71 cm in front of a 19-in. CRT monitor (width 36 cm; height 27 cm), which had a refresh rate of 100 Hz and a resolution of 1,024 × 768 pixels. Their eye movements were recorded with a sampling rate of 1,000 Hz using a video-based Eye-Tracker (EyeLink 1000; SR Research, Ontario, Canada). In all participants, the right eye was used for monitoring eye gaze. Participants’ heads were stabilized by a chin and forehead rest. Responses were recorded through a standard keyboard and mouse. Experiment Builder Software (SR Research) was used for controlling stimulus presentation and response collection.

**Figure 1.**
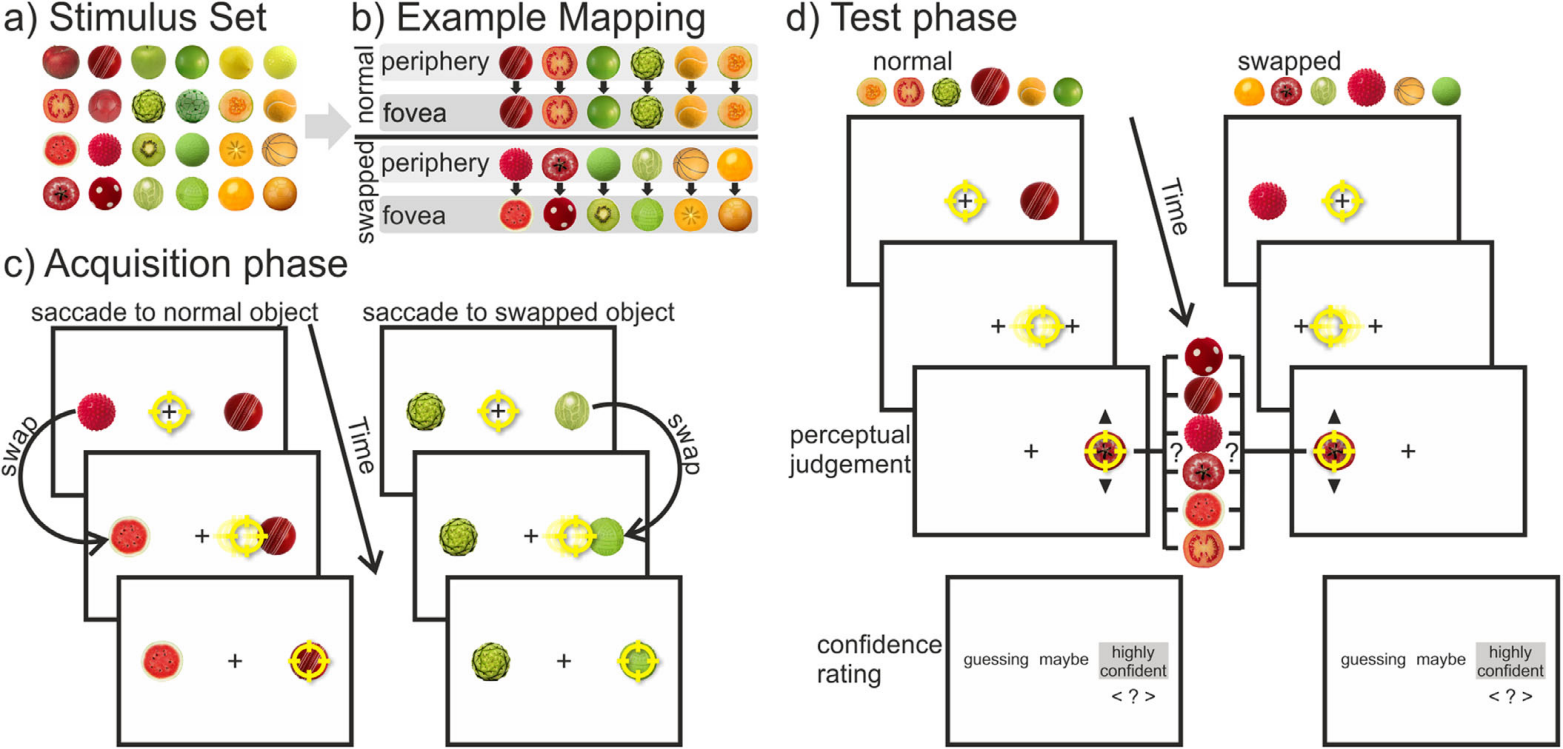
Design of Experiment 1. (a) Entire stimulus set, from which 18 stimuli were selected for each participant to create a unique mapping. (b) Example mapping for one subject shows the fixed mapping between the peripheral (i.e., presaccadic) and foveal (i.e., postsaccadic) view of the stimuli in the acquisition phase. There were six objects with the “normal” status that stayed the same and six objects with the “swapped” status that changed category during the saccade (half of them from fruit to ball, the other half from ball to fruit). (c) Structure of a trial in the acquisition phase: Participants could saccade freely to either of two objects. During the saccade, one of the objects was swapped out for an object of the opposite category. (d) Structure of a trial in the test phase: Participants had to saccade to the peripheral target, which was replaced by a fixation cross as soon as the eye movement started. After the saccade, participants could go through the objects one at a time via button press and had to choose which of them they had perceived presaccadically. Afterward, they also made a confidence judgment. Note for (c) and (d), stimuli are not drawn to scale. The yellow circle represents the gaze position.

The entire stimulus set consisted of 24 noncopyrighted pictures taken from the Internet of fruits and balls (see Figure 1a). They were chosen as stimuli through a pretest conducted with 18 other subjects. In this pretest, the stimuli were unambiguously categorized into three colors (red, green, yellow) and into two object categories (balls and fruits/vegetables). That means that all pictures in each subgroup were very similar within the low-level features of color and shape. For each participant, a unique mapping with 18 out of the 24 stimuli was created (see Figure 1b). Six objects (three balls and three fruits; two objects in each color category) were chosen as the “normal” objects. These would not be changed throughout the trials. Additionally, there were 12 objects chosen as the “swapped” objects. Half of them would only be presented in the periphery prior to a saccade, and the other half would only be seen foveally after the saccade. Moreover, there was a fixed mapping of the objects: One specific object was swapped out for another specific object within the same color category but from the opposite object category during the saccade. In half of the cases, the balls changed to fruits, and in the other half, fruits became balls. Again, there were two pairings in each color. In the end, this resulted in a randomly chosen but fixed item mapping per participant that was carefully counterbalanced across subjects to prevent any confounds with individual items in the set. The stimuli had a diameter of 1.6° and were presented at a horizontal eccentricity of 9° from the center. The central fixation cross was a black “+” (0.3° × 0.3°, line width of 2 pixels), and the background was white.

#### Procedure and design

The experiment was separated into an acquisition and a test phase. Each phase started with a 9-point grid calibration.

In each trial of the acquisition phase (see Figure 1c), participants first had to look at a central fixation cross for a variable time duration of 500 to 1,000 ms. Afterward, two different objects were presented on the left and right side, with the sides being randomized. Both were from the same color and object category, but one was from the fixed status category “normal”, and the other one was from the status category “swapped”. The participants’ task was to look at either one of the objects, the choice being their own. They were instructed, though, to look at both object categories, which they perceived foveally after the saccade, equally often. Furthermore, they should avoid fixed patterns (i.e., looking “left-right-left-right” or “fruit-ball-fruit-ball”). During the saccade, one of the objects (the one with the “swapped” status) was swapped out for its fixed counterpart of the same color but opposite object category. The other object (with the “normal” status) stayed the same during the saccade. For this online saccade detection, the real-time gaze positions were used. The gaze crossing an imaginary boundary 1° around the fixation cross was assessed as the start of a saccade, and the stimulus was changed with the next screen refresh. Following the saccade, the objects remained visible for an additional 250 ms. Then there was an intertrial interval of 1,500 ms in which a blank screen was presented. The training phase consisted of five blocks of 48 trials. After each block, participants received feedback on how often they looked at each object category.

Each trial in the test phase (see Figure 1d) started again with the fixation of a centrally presented cross whereupon one target object randomly appeared either on the left or right side. It could be any of the objects that had been presented in the periphery during the acquisition phase (i.e., 1 out of 12 objects). Participants’ first task was to saccade as quickly and accurately as possible to the object. As soon as the start of the saccade was detected, the object was replaced by a fixation cross. The saccade-contingent changes were elicited by the same boundary criterion as in the acquisition phase. Consequently, participants only had a peripheral but never a foveal view of the object. Trials in which the saccade latency was over 350 ms were aborted, and a message reminding participants to look at the target more quickly was shown. Five hundred milliseconds after saccade completion, one randomly chosen test object (out of six possible ones) appeared at the previous target position. These objects were all the fruits and balls of the same color category as the peripherally presented target. That is, it could be any of the fruits and balls they had already seen either pre- or postsaccadically in the acquisition phase. Participants’ second task was to pick the object that they had perceived in the periphery prior to the saccade. Via pressing the up or down arrow keys, they could go through all six possible objects one by one. They then submitted their final choice by pressing the Enter key. After each trial, they also rated how confident they were in their decision. The three choices they had were “guessing” (= –1), “maybe” (= 0), and “highly confident” (= 1). In total, the test phase consisted of 192 trials that were run in four blocks of 48 trials. Each block was composed of a factorial combination of two target locations (left vs. right), 12 target objects (factorial combination of two object statuses [normal vs. swapped], two object categories [fruits vs. balls], and three colors [red, green, yellow]), and two repetitions for each combination, presented in random order.

#### Data analysis

In the offline analysis, saccade onsets and offsets were identified by the EyeLink parser using a velocity criterion of 30°/s and an acceleration criterion of 8,000°/s^2^. Single trials from both the acquisition and test phase were excluded from the analysis if (a) saccades were anticipatory (latency < 80 ms) (B. Fischer et al., 1993; Wenban-Smith & Findlay, 1991), (b) gaze deviated by more than 1° from the display center at the time of saccade onset, (c) saccadic landing position deviated by more than 2° from the target position, or (d) saccadic latency was longer than 1,000 ms during acquisition or 350 ms during the test phase. With these criteria, 12.5% of all acquisition trials and 11.8% of all test trials were discarded from analysis. The significance criterion was set to *p* < 0.05 for all analyses. Statistical *t* tests are two-sided unless otherwise stated.

For the analysis of the test phase, different judgment errors were considered. First, judgment errors in general contained all judgments in which the picked object differed from the presented stimulus. Second, category errors were errors in which an object of the opposite category was selected (e.g., a ball was presented, but a fruit was selected). Third, for category errors, it was differentiated whether the incorrectly chosen object was the associated object (i.e., the object it was swapped out for during the saccade in the acquisition phase) or not.

### Results

#### Acquisition phase

Participants looked at the normal objects and at the swapped objects equally often (50.2% vs. 49.8%, respectively), *t*(23) = 0.224, *p* = 0.825, *d* = .046. They also followed the task and looked at the fruits and balls equally often (49.4% vs. 50.6%, respectively), *t*(23) = –1, *p* = 0.328, *d* = .204. The median saccade latencies per participant for the normal objects (*M* = 273.6 ms, *SD* = 113.7 ms) and the swapped objects (*M* = 274.8 ms, *SD* = 126.9 ms) did not differ significantly, *t*(23) = –0.168, *p* = 0.868, *d* = .034. The swapping of the objects (from fruit to ball or vice versa) occurred on average 26.7 ms (*SD* = 3.5 ms) after saccade onset. The mean saccade duration was 51.1 ms (*SD* = 10.2 ms).

#### Test phase

Previous studies (Herwig & Schneider, 2014) showed no differences between the presentation sides (left vs. right), and thus data were collapsed across this factor.

**Figure 2.**
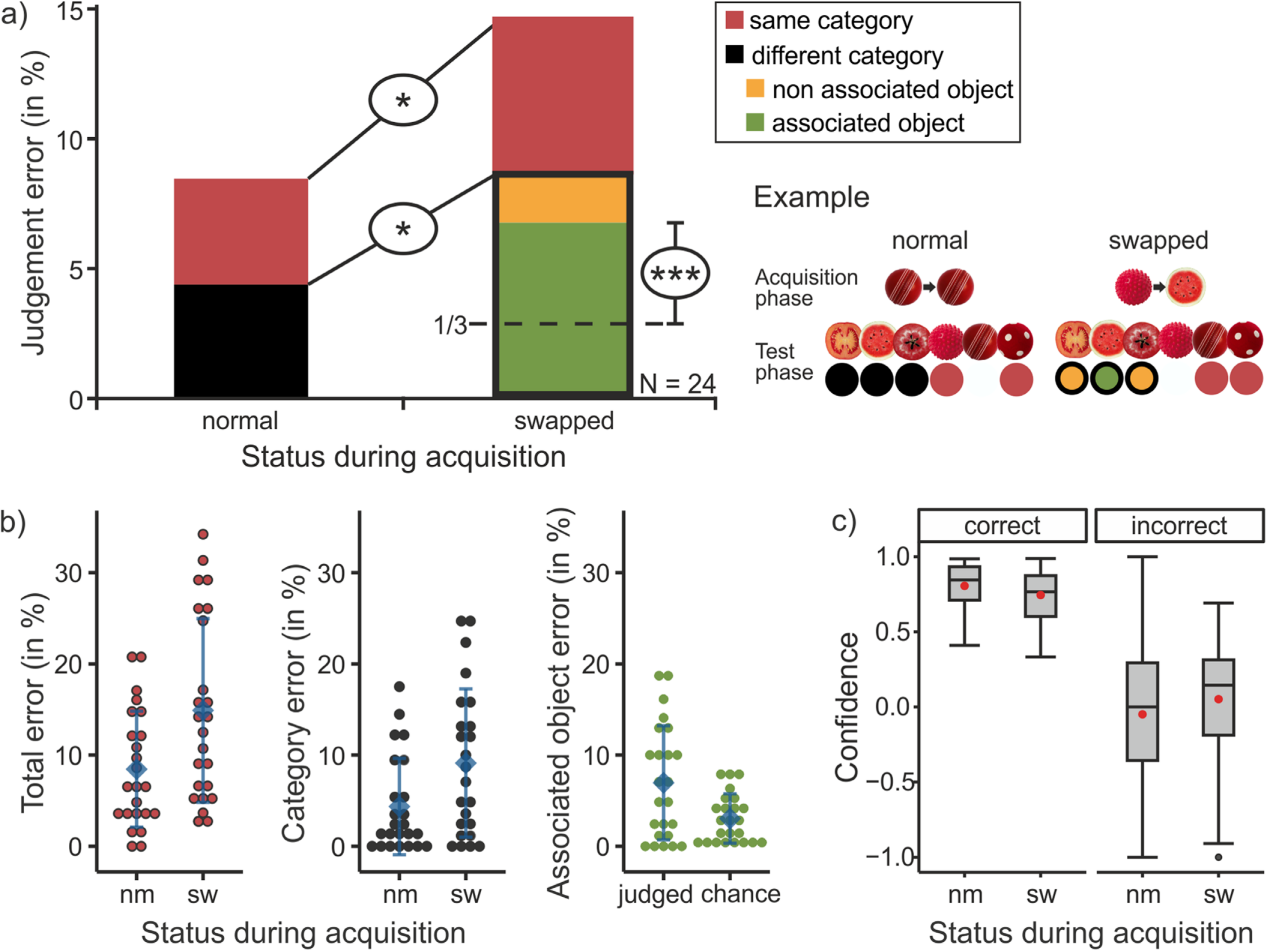
Results of Experiment 1. (a) Mean judgment errors (in percentages) in the test phase dependent on the object status during the acquisition phase (normal vs. swapped). Colors differentiate between the different kind of error types (the characteristics of the incorrectly chosen response). Next to the bar chart, one example is depicted: how the objects appeared in the periphery and the fovea during the acquisition phase, the possible responses that could be given when this object was presented during the test phase, and the kind of error this resulted in (color coded the same as in the bar chart). (b) The three comparisons that were made (and depicted in (a) via the black ellipses) depicted again with individual data points. On the left, the total percentage of judgment errors and in the middle the percentage of category errors are shown as a function of the status. The right graph shows how often the associated object was (falsely) chosen for the swapped objects (= “judged”) compared to what would be expected according to chance (one third of the different category rate). The blue diamond shows the respective mean, and the error bars represent the standard deviation across participants. For (b) and (c), “nm” is short for normal, and “sw” is short for swapped. (c) Boxplots of the confidence ratings dependent on whether the response was correct (left) or incorrect (right) and the status of the object during the acquisition phase (normal vs. swapped). The confidence rating ranged from “guessing” (= −1), “maybe” (= 0), to “highly confident” (= 1). Red circle: mean confidence rating.

For the analysis of the judgment errors (see Figure 2a), we calculated the percentage of trials in which errors occurred for each participant and report the average across participants. The analysis (also see Figure 2b, left plot) revealed that participants picked a wrong object significantly more often when a swapped object (i.e., one that was changed during the acquisition phase) was presented in the periphery (*M* = 14.9% of trials, *SD* = 10.1%) compared to when a normal object was presented (*M* = 8.5% of trials, *SD* = 6.4%), *t*(23) = –2.635, *p* = 0.015, *d* = .538. Within the trials in which errors occurred, it is possible to differentiate between choices within the same or a different category than the presented object. Category errors (also see Figure 2b, middle plot), that is, choosing an object of the wrong category, occurred more often when a swapped object was presented (*M* = 9.1% of trials, *SD* = 8.1%) than when a normal object was presented (*M* = 4.4% of trials, *SD* = 5.3%), *t*(23) = –2.395, *p* = 0.025, *d* = .489. Within the category errors that occurred when a swapped object was presented, a further differentiation was made between trials in which the associated object and trials in which one of the two nonassociated objects was chosen. The frequency with which the associated object was chosen was tested against one third of the category error frequency (chance rate). This (also see Figure 2b, right plot) showed that the associated object was chosen significantly more often (*M* = 7.0% of trials, *SD* = 6.3%) than the chance rate would allow, *t*(23) = 4.496, *p* < 0.001, *d* = .918.

The confidence ratings (see Figure 2c) were analyzed as a function of the two within-subject factors of object status during acquisition (normal vs. swapped) and the correctness of participants’ object judgment (correct vs. incorrect). Two subjects had to be excluded because they did not have ratings for all factor combinations. The repeated-measures analysis of variance (ANOVA) revealed a significant main effect of the judgment correctness, *F*(1, 21) = 122.804, *p* < 0.001, η_G_^2^ = .563. Trials in which a correct response was given received a significantly higher confidence rating than trials in which an incorrect response was given. The main effect of object status, *F*(1, 21) = 0.231, *p* = 0.636, η_G_^2^ = .002, and the interaction effect of status and correctness were not significant, *F*(1, 21) = 1.909, *p* = 0.182, η_G_^2^ = .013.

The median saccade latencies for the normal objects (*M* = 154.5 ms, *SD* = 23.5 ms) were slightly lower than those of the swapped objects (*M* = 158.7 ms, *SD* = 27.1 ms) during the test phase, *t*(23) = –2.220, *p* = 0.037, *d* = .453. The removal of the objects occurred on average 28.3 ms (*SD* = 3.4 ms) after saccade onset. The mean saccade duration was 50.3 ms (*SD* = 6.4 ms).

## Experiment 2: Gender of faces

### Materials and methods

#### Aim

In the second experiment, we tested whether a biasing effect due to transsaccadic predictions could also be shown for the gender of faces. During the acquisition phase, for some participants, a male face was changed to a female face (both taken out of a morphed sequence of faces that ranged from male to female), and another face (taken out of a different face sequence) stayed the same. For other participants, the faces changed in the opposite direction. In a following test phase, participants were presented with any of the morphed faces out of the sequences and had to judge how they perceived them. We hypothesized that perception of the faces would be biased toward the previously associated foveal gender. That is, for example, participants would perceive the presented face as more female if it was in the sequence that was changed from male to female during acquisition.

#### Participants

Sixteen participants (nine female, six male, one diverse) participated in Experiment 2. They were aged between 18 and 30 years (*M* = 23.44, *SD* = 3.18). There was no overlap in these participants with the cohort from Experiment 1. Participation criteria as well as the ethical standards were the same as in the first experiment.

#### Apparatus and stimuli

The same setup as in Experiment 1 was used. The only difference was that for Experiment 2 PsychoPy3 (Peirce, 2007) was used for controlling stimulus presentation and response collection.

**Figure 3.**
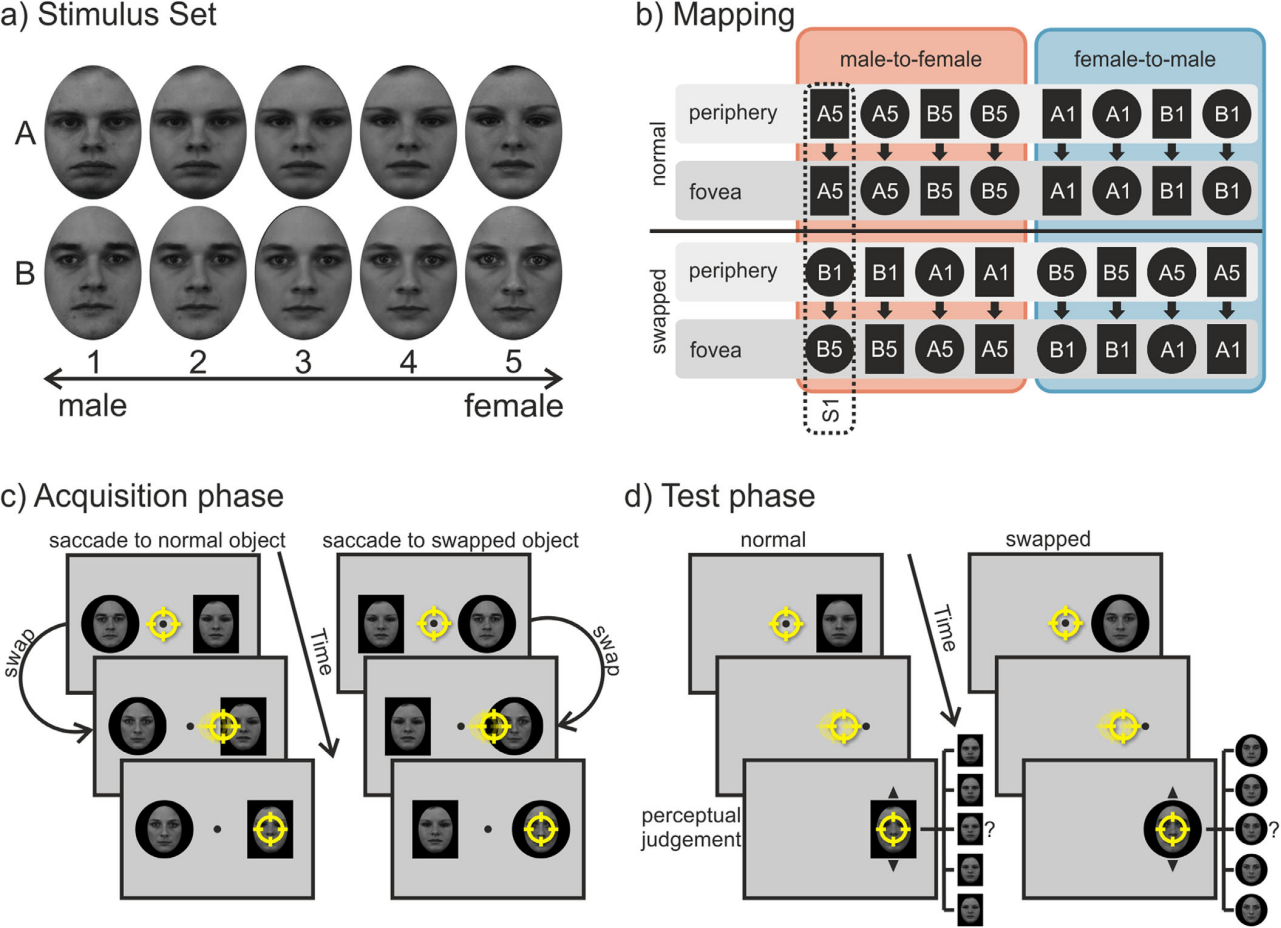
Design of Experiment 2. (a) Entire stimulus set containing two face sequences (A and B) ranging from male to female. The numbers indicate the gender score. (b) Schematic mapping of all the different counterbalanced stimulus combinations used in the acquisition phase. The black circles and rectangles picture the fixed background shape behind the face image, and the letters and numbers represent the specific face. For each subject (each vertical column, one example is indicated by the dashed line), there was a fixed mapping between the peripheral (i.e., presaccadic) and foveal (i.e., postsaccadic) view of the faces. There was one face with the “normal” status that stayed the same and one face with the “swapped” status that changed either from male-to-female (outlined by the red box) or from female-to-male (outlined by the blue box) during the saccade. (c) Structure of a trial in the acquisition phase: Participants could saccade freely to either of two objects. During the saccade, one of the faces was swapped out for a face of the opposite gender. (d) Structure of a trial in the test phase: Participants had to saccade to the peripheral target, which was replaced by the fixation point as soon as the eye movement started. After the saccade, participants could go through the faces one at a time via mouse wheel and had to choose which of them they had perceived presaccadically. Note for (c) and (d), stimuli are not drawn to scale. The yellow circle represents the gaze position.

The stimulus set consisted of two sequences of gray-scaled images of faces with a neutral expression (see Figure 3a). Each sequence consisted of five face morphs that ranged from female to male. The original four faces (two male, two female) were taken from the Karolinska Directed Emotional Faces databank (Lundqvist, Flykt, & Öhman, 1998). Morphs between the two pairings of female and male faces were generated using FantaMorph (Abrosoft, https://www.abrosoft.com/). That is, each sequence was created from one specific male-female couple as the extremes (male face = gender score of 1; female face = gender score of 5) and three morphed faces in between. All faces were equated in mean luminance and contrast using the SHINE toolbox (Willenbockel et al., 2010). The images of the faces were cropped by an oval aperture (2.9° wide and 4.0° high) and presented on top and in the center of either a black rectangle (3.9° wide and 5.0° high) or a circle (5° diameter). The background shapes (rectangle or circle) served as a peripherally unambiguous discriminative feature between the two face sequences for the associative learning. For each participant, the combination of the face sequence (the specific couple) and background shape (rectangle or circle) was fixed for the entire experiment but was counterbalanced between subjects. The central fixation stimulus was a circle with a diameter of 0.25°, and the invisible circular fixation area around it had a diameter of 3°. Stimuli were presented on a gray background.

#### Procedure and design

Again, the experiment consisted of two phases: an acquisition phase and a test phase. Each phase started with a 9-point grid calibration.

At the beginning of each trial in the acquisition phase (see Figure 3c), participants had to look at a fixation point in the center of the screen. After a variable fixation interval of 500 to 1,000 ms, two faces (one from each sequence) appeared 6° left and right of the center. The sides on which the faces appeared were alternated pseudo-randomly. Participants’ task was to saccade to one of the two faces. The choice was voluntary, but their aim was to look at both faces (easily distinguishable by their fixed background shape) equally often throughout the experiment. Again, they were also instructed to avoid fixed viewing patterns (e.g., left-right-left-right). During the saccade, one of the faces changed in the dimension of gender. It either changed from male to female (Face 1 changed to Face 5) or from female to male (Face 5 changed to Face 1). The other face stayed the same during the saccade. The change direction and for which of the two sequences (face-shape combinations) it occurred stayed constant for each participant but was counterbalanced across subjects (see Figure 3b). That is, participants always saw two faces that differed in their gender in the periphery, but they saw faces with the same gender in the fovea. After the end of the saccade, the image of the faces remained visible for additional 250 ms followed by an intertrial interval of 1,500 ms with only a blank gray screen. The acquisition phase consisted of 240 trials run in five blocks of 48 trials. After each block participants received feedback on how often they had looked at each face.

In the test phase (see Figure 3d), after a fixation interval of 500 to 1,000 ms, one face appeared either on the left or right side. Subjects’ task was to saccade as quickly and accurately as possible to this face. As soon as the fixation area was left, indicating the start of a saccade, the face was replaced by the fixation point. This ensured that subjects only had a peripheral view of the face and never a foveal one. After the saccade ended, the fixation point stayed visible for additional 500 ms before being replaced by a randomly picked face out of the same sequence that was peripherally shown. Participants then had to respond which face they had perceived in the periphery prior to the saccade by going through the sequence of faces (differing in gender) via the mouse wheel and submitting their answer via a keypress on the keyboard. All 10 faces of the two sequences were presented during the test phase but in different frequencies. In two thirds of the trials, Face 3 (the median gender) in the sequence was presented, because possible judgment biases in both directions could be measured equally well. In the other one third of the trials (catch trials), the rest of the faces in the sequence were shown with equal frequencies to ensure that participants would not notice the uniformity of the faces’ gender. Trials in which participants did not look at the target quickly enough (latency > 500 ms) or precisely enough (saccade ended outside of a 2.5° radius of the target) were aborted and participants received an error message asking them to perform the eye movement quicker or more precisely. These trials were repeated at the end in a randomized order. Participants had more time to saccade to the target than in the first experiment, because the task of identifying the presented faces was more difficult (experience while piloting). Originally (without redoing trials), the test phase consisted of 192 trials, which were run in four blocks of 48 trials. Each block counterbalanced the factors of target locations (left vs. right) and target objects (ten different faces) while presenting them in a pseudo-random order. After the test phase, participants were questioned whether they had noticed any changes during the acquisition phase.

#### Data analysis

The same offline saccade detection criterions as in the first experiment were used. Single trials were excluded from the analysis if (a) the saccade latency was anticipatory (latency < 80 ms), (b) saccade latency was too large (latency > 500 ms in the test phase or 1,000 ms in the acquisition phase), (c) the saccade to the target started outside of a 1.5° radius of the fixation point, (d) end points of the saccade were outside of a 2.5° radius of the center of the target, and (e) the swap of the changed object in the acquisition phase or the disappearance of the target object in the test phase did not occur during the saccade. The latter occurred in 1.4% of trials in both the acquisition and test phase. With all the abovementioned criteria, on average, 5.3% of trials were discarded in the acquisition phase and 3.0% in the test phase. In the analysis of the test phase, only the trials in which faces with a gender Level 3 in the sequence were shown as the test item in the periphery were included. The significance criterion was set to *p* < 0.05 for all analyses.

### Results

#### Acquisition phase

Participants looked at the normal faces in 48.9% and at the swapped faces in 51.1% of trials. We tested whether these proportions differed significantly from the instructed 50% for each subject separately, which was never the case, χ^2^s < 0.987, *p*s > 0.321. The median saccade latencies for the normal faces (*M* = 179.9 ms, *SD* = 29.9 ms) and the swapped faces (*M* = 176.6 ms, *SD* = 30.9 ms) did not differ significantly, *t*(15) = 1.362, *p* = 0.193, *d* = .340. The swapping of the faces occurred on average 21.8 ms (*SD* = 3.5 ms) after saccade onset. The mean saccade duration was 47.8 ms (*SD*= 8.0 ms).

#### Test phase

For all analyses, only trials in which faces of Gender 3 in the sequence were presented as a test item in the periphery were used and the catch trials were discarded. Again, data were collapsed across presentation sides (left vs. right).

**Figure 4.**
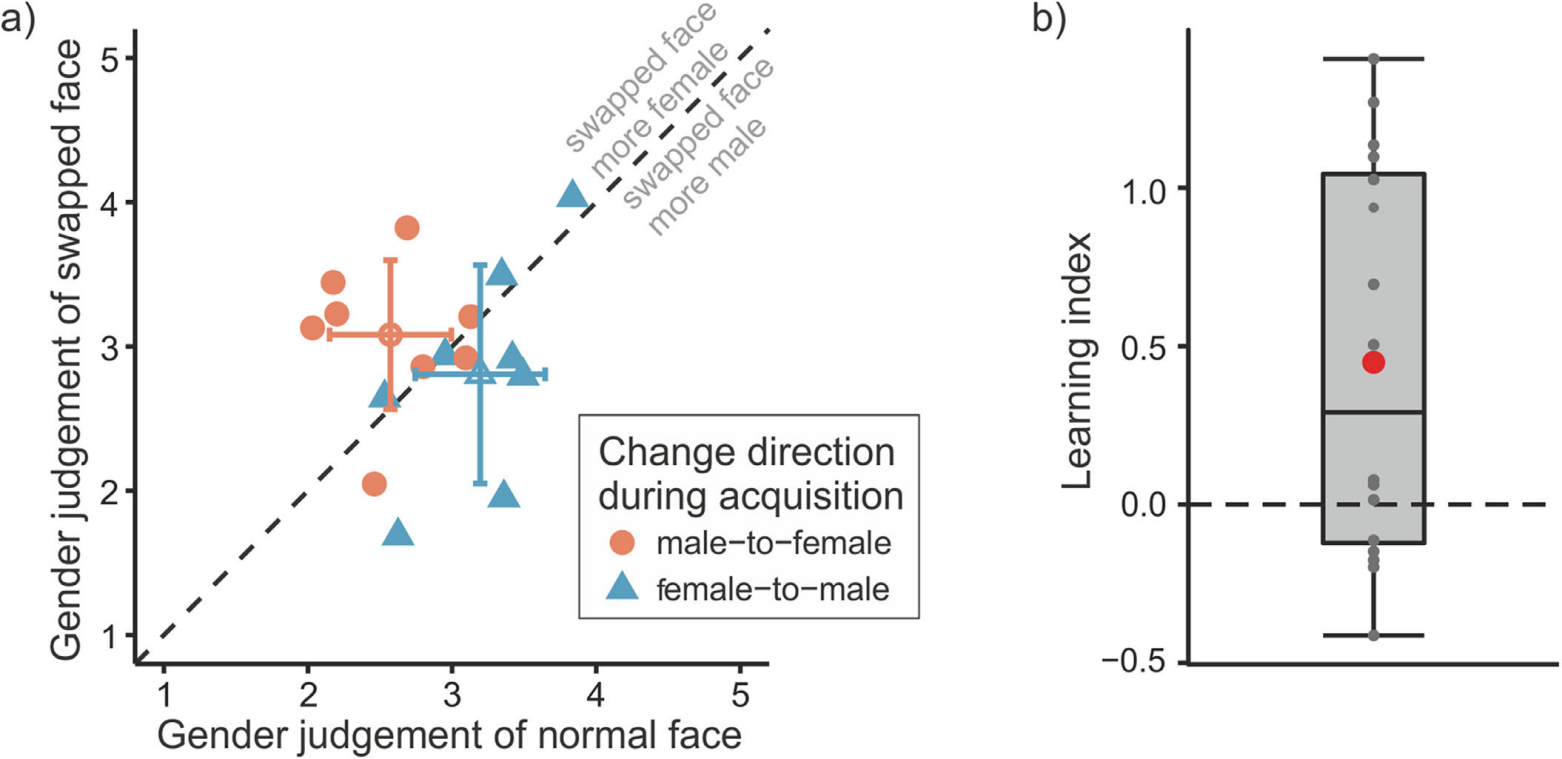
Results of Experiment 2. (a) Mean judgments of the gender of the swapped face as a function of the gender judgment of the normal face for each participant separately (filled shapes) and averaged across participants (open shapes). For the latter, the error bars represent the standard deviations of the means. Color and shape distinguish the change direction of the swapped face during the acquisition phase. Gender scores range arbitrarily from 1 (= male) to 5 (= female). (b) Boxplot of learning indices (i.e., judgment differences between normal and swapped faces). Positive values indicate a judgment bias toward associated foveal inputs of the acquisition phase. Overall, the learning index was significantly greater than zero. Gray dots indicate the learning indices for each participant; the red circle shows the mean learning index.

Figure 4a plots the gender judgments of the normal faces against the judgments of the swapped faces for each participant and change direction. Here, a separation of the data points by the equality line according to the change direction can be seen, indicating a judgment bias toward the associated foveal input during the acquisition phase. It is evident for most participants, though, there are also some data points on or close to the equality line, indicating that these participants did not show any (strong) effect.

As a measure for the effect of the transsaccadic learning on the gender judgments, a learning index was computed for each participant by subtracting the average gender judgment of the normal face from the judgment of the swapped face for the male-to-female group and subtracting the average gender judgment of the swapped face from the judgment of the normal face for the female-to-male group. Thus, a positive value indicated a judgment bias of the changed object in the direction of previously associated foveal input, and a negative value indicated a judgment bias in the opposite direction (for a related procedure, see also Herwig & Schneider, 2014; Paeye et al., 2018). These learning indices were significantly greater than zero, *t*(15) = 2.883, *p* = 0.011, *d* = .721, which is depicted in Figure 4b.

Median saccadic latencies per participant of the test phase were analyzed as a function of the within-subjects factor status in the acquisition phase (normal vs. swapped) and the between-subjects factor change direction (male-to-female vs. female-to-male). A 2 (status) × 2 (change direction) mixed ANOVA did not reveal any significant main or interaction effects, *F*s < 1.407, *p*s > .255, η_G_^2^ s < .041. Averaged across the factors of status and change direction and across participants, the mean of the median saccade latencies in the test phase was 137.6 ms (*SD* = 20.8 ms). The removal of the peripherally presented faces occurred on average 21.8 ms (*SD* = 3.4 ms) after saccade onset. The mean saccade duration was 46.1 ms (*SD* = 6.1 ms).

#### Postsession debriefing

The postsession debriefing revealed that 15 out of 16 participants did not notice the change of gender during the acquisition phase. One participant supposedly noticed the change, but the reported change direction was not correct.

## Discussion

Several recent studies indicate that the visual system predicts visual features across saccades based on learned transsaccadic associations between peripheral and foveal input. However, the stimuli that were used in these studies were simple and artificial, and predictions were made only within one feature dimension: for example, shape (Herwig et al., 2015; Köller et al., 2020; Paeye et al., 2018), size (Bosco et al., 2015; Valsecchi et al., 2020; Valsecchi & Gegenfurtner, 2016), or spatial frequency (Herwig & Schneider, 2014; Herwig et al., 2018). The two experiments of the present study extended these findings by demonstrating that humans can also make transsaccadic predictions about more complex stimuli (fruits/balls and faces), which are then reflected in a biased perception. Furthermore, Experiment 1 showed that numerous new associations can be learned within a short time span.

In the first experiment, transsaccadic associations between balls and fruits were established during the acquisition phase. In the test phase, participants were able to identify the correct object that was peripherally presented to them in the majority of cases. But sometimes (in 10–15% of the cases), they made judgment errors. These errors occurred more often for objects that had been previously swapped during the acquisition compared to objects that had not been swapped. Furthermore, participants chose the wrong category significantly more often for objects that had been swapped compared to the not swapped objects. Importantly, these category errors occurred mainly because participants chose with a higher-than-chance rate exactly the transaccadically associated foveal counterpart of the presented peripheral object. This latter finding might shed some light on the kind of processing that underlies the transsaccadic learning and predictions. One possibility is that certain rules are learned that govern how peripheral input needs to be processed to predict the foveal percept. This process would be rather computationally intensive, but one would expect an easy transfer to other situations. The other possibility is that individual object instance associations are stored and then retrieved during perception. This process would be quite memory intensive, and transfer to novel situations should be more difficult. The finding that category errors occurred mainly because participants chose the previously associated foveal counterpart suggests that associations were not generalized into a rule about category changes (e.g., “fruits change to balls during the saccade”). Instead, individual object instances were associated, and hence the learning in Experiment 1 can be considered rather object specific than rule based. This resembles perceptual learning, which has also been argued to occur in an object-specific mechanism (Furmanski & Engel, 2000). Admittedly, such a processing, in which stimulus instances are all stored separately, is highly memory intensive (Dill & Fahle, 1997), especially considering the vast number of different objects that can be encountered in everyday life and for which in conclusion transsaccadic associations are potentially memorized as well. Nevertheless, it seems possible as long-term associative memory has an estimated capacity of several thousand associations (Voss, 2009). Other studies about visual long-term memory further demonstrate its massive capacity for storing even details of objects (Brady, Konkle, Alvarez, & Oliva, 2008) and suggest that there is virtually no limit for the retention of item-specific visual information (Standing, 1973). Another theory that potentially fits well with this object-specific and memory-intensive learning is the instance theory of automatization (Logan, 1988). It presents a learning mechanism called automatization in which algorithmic processing transitions into memory-based processing through the encoding, storing, and retrieval of separate stimulus encounters.

Experiment 1 further revealed that participants’ confidence was not affected by the status of the presented object (i.e., whether it was a normal or a swapped object). Thus, learning new transsaccadic associations for some items did not result in an experienced difference in the confidence perception of them. Confidence reflected only the correctness of responses: Participants were more confident when they made correct responses and less confident when they made incorrect responses. This fits well to others studies showing a strong correlation between confidence and performance (Kotowicz et al., 2010) or even between participants’ confidence and the precision of their working memory for items (Rademaker, Tredway, & Tong, 2012). There are different possibilities of why participants give a low confidence rating. The first is that they temporarily do not pay attention and consequently must guess the answer. Participants are aware of this and are therefore not confident in their answer. This would result in equal frequencies between all response options. The second scenario would be that what they perceived was not unambiguous to them and therefore they cannot decide between certain items. Again, this results in participants having low confidence in their response, but in this case, they do not decide randomly between all options. Because the results showed that participants picked the associated item with a higher rate than if they had simply guessed, the latter scenario must have been true (at least in some cases). This further demonstrates that participants likely perceived a mixture of the peripherally presented item and the prediction associated with it. And in some of these ambiguous cases, they relied more on the latter one.

The second experiment tested whether transsaccadic predictions can also be learned for more complex stimuli in an experiment with a more metric response judgment. With this type of response, it is possible to address the question to what extent perception is biased toward previously associated foveal input. Therefore, Experiment 2 used morphed pictures of human faces that ranged from female to male. The results showed that participants can learn transsaccadic changes in the gender of faces. After the acquisition phase, their perception of the gender of peripherally presented faces was biased in the direction of the learned foveal association. That is, participants for whom the faces of the changed sequence changed from male to female during acquisition perceived the median gender face of that sequence more female than that of the sequence in which no gender change occurred during acquisition. Conversely, participants for whom the transsaccadic change occurred from female to male during acquisition thereafter perceived the median face of the swapped sequence more male compared to that of the normal sequence.

Interestingly, the size of the biasing effect is comparable to previous studies, which used simple stimuli. Averaged across participants, the judgment difference between the normal and the swapped median face relative to the change size during acquisition reflects an 8.88% contribution of the newly acquired foveal prediction into the peripheral percept. That is exactly the maximum relative contribution that was found for shape changes in Köller et al. (2020). The fact that there were some participants who did not show any strong effect might be due to the difficulty of the task (evident in the large variances in the response data as well as in the reports of participants). The peripheral viewing time in our experiment was limited to 500 ms, which is longer than in previous experiments on simple visual features (350 ms) but still shorter than in other studies about face perception in which a method of adjustment was used (750 ms) (e.g., Liberman, Fischer, & Whitney, 2014). Thus, at least for some participants, more encoding time might have been necessary for these complex stimuli.

For the second experiment, the perceptual bias could also indicate an adaption-like effect. Each participant only saw three out of the four extreme faces (A1, A5, B1, B5) during the acquisition phase and might have adapted to these images. Consequently, the perception of the neutral face in the normal sequence would be biased away from the seen face and toward the unseen face. Multiple studies have shown this kind of aftereffect for faces. For example, faces are perceived as distorted in a direction opposite to the adapting distortion (Webster & MacLin, 1999), or gender perception of previously ambiguous faces is biased away from the adapting gender (Webster, Kaping, Mizokami, & Duhamel, 2004). Thus, this alternative explanation cannot be completely ruled out. However, in all these (and other adaption studies), the presentation duration of the adaptation stimulus was much longer (mostly several seconds to minutes) with additional repetitions (again often several seconds) before each test trial. In comparison, in our study, the presentation times were well below a second and the potential adaptation was also not repeated in the test phase. Leopold, Rhodes, Müller, and Jeffery (2005) could show that face identity aftereffects increase logarithmically with adaptation time, although their adapting times ranged from 1 to 16 s. It is therefore unlikely that our short presentation times led to an adaptation effect. Furthermore, studies with transsaccadic changes of object size with only a “swapped” object could also show a recalibration of perception toward the postsaccadic foveal association (Valsecchi et al., 2020; Valsecchi & Gegenfurtner, 2016).

Taken together, the visual system was able to learn or update multiple new transsaccadic associations for complex and more realistic stimuli within a short time frame. Predictions based on these associations were integrated presaccadically with the actual peripheral input resulting in a biased perception. This demonstrates that the human brain constantly keeps track of certain statistics of our environment to make accurate predictions about our surroundings. Of course, the stimuli used in the present experiments were far away from depicting natural scenes, but still, these complex stimuli are a step closer to realistic everyday objects. Therefore, our findings might be taken as a first tentative hint to the functional relevance of this transsaccadic prediction mechanism outside the laboratory.

Previous studies have shown that the prediction mechanism for peripheral stimuli does not depend on the execution of a saccade (Paeye et al., 2018; Valsecchi & Gegenfurtner, 2016). Instead, it has been suggested that the mechanism reflects a more general function of the visual system, which allows the prediction of detailed foveal information given coarse peripheral input. If predictions are made for multiple and complex objects in our periphery, it could lead to the impression of perceptual homogeneity in our field of view. Nevertheless, the predictive mechanism seems to profit from the saccadic eye movements as stronger biasing effects can be seen here compared to fixation conditions (Paeye et al., 2018). Possibly the prediction system is optimized for saccades, because they are the most common event that leads to the acquisition of peripheral-foveal associations.

The presented results further extend findings made in the study by Cox, Meier, Oertelt, and DiCarlo (2005), which showed predictable object confusions across the saccade for “greeble” stimuli. These are also complex but, in comparison to our study, very artificial und unfamiliar stimuli. The objects only changed slightly during the learning phase and did not change their semantic category like the stimuli in our experiments. Object predictions in their study were inevitably linked with the objects’ presentation side. This was not the case for the present study, where predictions were made based on the peripheral image irrespective of the presentation side. Furthermore, with our second experiment, we used a metric response mode, which allowed us to estimate the relative strength of the predictions. The study by Cox et al. (2005), on the other hand, used a same-different task, which leaves open the question how exactly the objects that were judged as “different” were perceived.

Previous studies have suggested that transsaccadic learning is very specific to its retinotopic location (Herwig et al., 2018) and that transsaccadic prediction is therefore likely to take place in low- or mid-level visual areas where a classical and finer retinotopy is prevalent (Gardner, Merriam, Movshon, & Heeger, 2008; Grill-Spector, 2003; Hadjikhani, Dale, Liu, Cavanagh, & Tootell, 1998). But this location specificity might have only occurred because these studies used simple stimuli that are represented in low- or medium-level visual areas. The complex stimuli that were used in the present study are also represented as semantic objects (fruits or balls) or faces in high-level brain areas like the inferior temporal (IT) cortex (Kravitz, Saleem, Baker, Ungerleider, & Mishkin, 2013; Tanaka, 1993). Consequently, it could be assumed that transsaccadic predictions about objects might also originate in these high-level brain regions where the objects are represented. Support for this idea can be found in a study by Li and DiCarlo (2008), in which, similar to the presented study, high-level objects where swapped out during the saccade. After enough experience, this led to a decrease in initial object selectivity of IT neurons (in the primate brain). Thus, a higher level of the ventral visual stream (e.g., Rousselet, Thorpe, & Fabre-Thorpe, 2004) was affected by the transsaccadic manipulation.

If transsaccadic predictions are based on high-level object representations, the question arises whether they would still be location specific. For example, the fusiform face area within IT, which shows an increased activity to the presentation of face stimuli (Kanwisher, McDermott, & Chun, 1997), has been described as a nonretinotopic region of the ventral stream (Halgren et al., 1999). Accordingly, it is presumable that predictions for faces (or other high-level representations) are not retinotopically location specific but generalize to other locations. This is something that could be investigated in the future.

On the opposite, assuming that the predictions must originate in these higher-level areas is not necessarily true. One cannot rule out the possibility that participants were able to identify or predict the objects based on certain low-level features within the complex stimuli. It is also conceivable that high-level areas govern the reweighting of primary cortex inputs, and these weights are changed during the learning process, as presented in a rule-based learning model for perceptual learning by Zhang et al. (2010). Thus, based on the current study, one cannot conclude whether the prediction was made before or after a semantic representation of the objects and faces was created. Hence, more research is needed to further differentiate when and where exactly in the brain the presaccadic integration of prediction and peripheral input takes place. From the neural study by Edwards, Vanrullen, and Cavanagh (2018), it is known that peripheral presaccadic stimulus information is available after the saccade and influences postsaccadic processing (e.g., congruent presaccadic input facilitates the processing). Huber-Huber, Buonocore, Dimigen, Hickey, and Melcher (2019) suggested that first a prediction about the target is generated, and then the integration of presaccadic and postsaccadic information takes place at around 50 to 90 ms after fixation onset, followed by a facilitated categorization. By using a similar methodology (combined electroencephalogram and eye-tracking), it might be possible to narrow down the timeline for the presaccadic processing even more.

## Conclusion

Our visual system constantly learns transsaccadic associations and makes predictions about features or object identities based on them. This has previously been shown for simple visual features such as shape, size, and spatial frequency. In the current study, we could extend knowledge about this presaccadic integration process by showing that humans can also learn transsaccadic associations for more complex visual stimuli (i.e., fruits, balls, and faces). Perception was biased toward the newly associated foveal percept of the stimuli. Moreover, our results suggest that multiple associations can be learned within a short time frame and that the process is object specific.
